# Book Review

**Published:** 1928

**Authors:** 


### Reviews of Books

**Some Principles of Diagnosis, Prognosis and Treatment.** By R. HUTCHISON, M.D., F.R.C.P. Pp. vi., 54. Bristol: John Wright & Sons Ltd. 1928. Price 2s. 6d.—Everything that Dr. Hutchison writes is worth reading, for he is able to interpret to us in a sane and concise manner the lessons of his great experience. The tract now in our hands is no exception to this rule. It is so interestingly written and excellently printed that it would make no bad present to give to a graduate of medicine about to begin his first resident appointment.

**A Shorter Anatomy.** By E. WOLFF, F.R.C.S. Pp. viii., 451. 130 illustrations. London: H. K. Lewis & Co. Ltd. 1927. Price 18s.—Under the title of *A Shorter Anatomy* Mr. E. Wolff has written a book which differs considerably from others. It is intended especially for students revising their anatomy for the final examinations, and the descriptions are given with a view to their practical application. The subject is presented in an unusual but interesting manner with an account of surface appearance, and a considerable number of useful descriptions, in the classified form so much appreciated by students. The book is written chiefly from the point of view of the surgeon, and the central nervous system and the thoracic viscera are scarcely mentioned. But, while there is a very useful chapter on ossification and epiphyses, and the joints are considered in some detail, there is no systematic account of osteology. Important points are stressed in regard to their practical application; but it would hardly seem necessary in a book of this type to give a measurement to one-eighth of an inch, for the distance of the sterno-clavicular joint from the middle line of the body. In the account of the organs of sense there is an excellent description of the anatomy of the eye; but in the chapter on the auditory apparatus there is no mention of the internal ear. On account of the attractive form in which the excellent subject-matter is presented in this book, it should prove a boon to those who wish to revise their knowledge of anatomy with the least possible effort.

**The Essentials of Medical Diagnosis.** By SIR THOMAS HORDER, Bart., K.C.V.O., M.D., F.R.C.P., and A. E. GOW, M.D., F.R.C.P. Pp. xx., 682. Illustrated. London: Cassell & Co. Ltd. 1928. Price 16s.—It is to be expected that a book coming from such a source should achieve a particularly high standard. The authors have succeeded in compressing into small volume a large amount of valuable information. The arrangement of the subject-matter is particularly well planned. Each section begins with a succinct account of the anatomy of the region such as is essential for the adequate examination of the patient. This is followed by an outline of the more important details of clinical pathology. The argument of the book is from the patient to the disease. The authors stress the value of absolutely systematic examination, and their general attitude towards accurate diagnosis is expressed in their introduction: "To know exactly what signs to look for in any particular case demands enough knowledge of the disease to enable the observer to have in his mind from the outset of his survey some tentative idea as to the nature of the case." He is warned, however, to keep this initial attitude nebulous, lest he become biased and attempt to fit in his observations with some preconceived notions. Where all is so good criticism is difficult. It is, perhaps, disappointing to find diabetes dismissed in a single page. Of the chapter on diseases of the lungs many would question the authors' view of the causation of Grocco's triangle of dulness. The nomenclature of adventitious sounds in the lungs is clear, but does not entirely conform to that adopted by others; and impresses on one the need for a fixed standard nomenclature. Of the sections on clinical pathology, the one dealing with the examination of the urine is particularly useful. The book is full of interest, and as a supplement to one of the standard text-books on medicine may be strongly recommended.

**Handbook of Sanitary Law.** By B. B. HAM, M.D., D.P.H. Tenth Edition. Pp. xxxii., 284. London: H. K. Lewis & Co. Ltd. 1928. Price 6s. 6d.—The tenth edition of this book is brought up to date by the inclusion of all recent sanitary legislation. It still manages to compass within 270 pages a complete synopsis of all the law which is necessary for a D.P.H. student to know, without sacrificing clarity to brevity. While due space has been given to the Milk and Dairies Order, 1926—the most important addition—the book still retains its conveniently portable form.

**Synopsis of Physiology.** By A. R. SHORT, M.D., B.Sc., F.R.C.S., and C. I. HAM, M.B., B.Ch. Pp. vi., 258. 27 illustrations. Bristol: John Wright & Sons Ltd. 1927. Price 10s. 6d.—In the preface of the last addition to the already famous "Synopsis" series the authors state that their object in writing the book has been "to give a full summary of modern physiology, particularly human physiology, in a small compass." This object they have successfully attained; though in a book for medical students, who will later proceed to clinical work, it seems unfortunate that more attention has not been paid to physiology as applied to clinical medicine, and also to the physiological information gained from recent clinical investigations. For example, in the chapter dealing with the heart no mention can be found of fibrillation or of circus movement; and in the chapter on gastric function the normal duodenal reflex, demonstrated by MacLean, which has so important a bearing on the interpretation of a fractional test meal, has escaped inclusion. But these are minor points, and the chapters on the nervous system are particularly good. Dealing, as they do, with very controversial subjects, the known facts and the generally accepted current ideas are stated clearly and succinctly, and should be of great value to students who may have been confused by the apparent contradictions of a larger text-book. The book should prove popular, not only with students, but also, as the writers suggest, with lecturers who wish to make a rapid revision of the essentials of their subject.

**The Meningiomas.** By H. CUSHING, C.B., D.S.M., M.D., LL.D. Pp. vi., 53. 28 illustrations. Glasgow: Jackson, Wylie & Co. 1927. Price 2s. 6d.—This monograph records with singular clarity and beauty of diction how science has defined yet another clinico-pathological entity (meningiomas of the olfactory groove) from the divers multitude that are embraced under the heading of cerebral tumour. Not only does the author fire the diagnostic zeal of the clinician, but whets the technical appetite of the surgeon. He writes in glowing terms of a new boon to cerebral surgery in the form of diathermy and similar coagulating electric currents. Turning from this highly-specialised subject to the wider realm of the surgery of neoplasms in general, he foreshadows a time when the surgeon will forsake his scalpel and work with cutting electrodes in a bloodless field. He regards electro-surgery as an advance of a magnitude little less than the Listerian discovery.

**Rheumatic Diseases.** Proceedings of a Conference held at Bath, May, 1928. Pp. xi., 292. Published by the Hot Mineral Baths Committee of the Bath City Council, 1928. The editing of this volume must have been a difficult task. Those who sat through the Bath Conference were almost overpowered by the complexity of the problem which was discussed, and although those responsible for its organisation achieved much in the direction of simplification, much remained to be done. To Dr. R. G. Gordon and his colleagues of the Organising Committee we offer our respectful congratulations on this highly satisfactory consummation of their undertaking. The book should prove of permanent value as an expression of modern opinion on a subject that is of interest to every practitioner of medicine.

**Neoplasms of the Bladder.** By L. R. FIFIELD, F.R.C.S. Pp. xi., 94. 12 illustrations. London: H. K. Lewis & Co. Ltd. 1928. Price 7s. 6d.—This small monograph is the essay for which the Buckston-Browne Prize was awarded by the Harveian Society, and has been published just after the untimely death of the author from a street accident. The most important part of the work consists in the experimental observations made on the subject of transplantation of ureters into the bowel, this being illustrated by coloured figures. The author concludes that bilateral nephrostomy and ureterostomy are the safest and the most generally applicable methods of diverting the urine in cases of carcinoma of the bladder; that Stiles and Coffey's methods of uretero-intestinal anastomosis are equally good, but both attended by grave risks, nearly 60 per cent. developing ascending infection, and about 30 per cent. stricture of the lower end of the ureter. The book is well and clearly written and affords a good summary of the subject.

**The Injection Treatment of Varicose Veins.** By A. H. DOUTHWAITE, M.D., M.R.C.P. Third Edition. Pp. x., 51. London: H. K. Lewis & Co. Ltd. 1928. Price 4s.—Varicose veins, ulcers and eczema are with us still as frequent ills, and in this second edition of his book Dr. Douthwaite deals most comprehensively with their treatment by injection. If we make haste to follow it, ours will be the privilege of providing the public with a simple cure for one of the really common ills.

**Applied Physiology.** By S. WRIGHT, M.D., M.R.C.P. Second Edition. Pp. xxvi., 510. Illustrated. London: Oxford University Press. 1928. Price 18s.—In the second edition of this already popular work the author has more than maintained the high standard of excellence set in the first edition. The whole book has been thoroughly revised and brought up to date, no small task considering what rapid advances have been made in physiology over a large front in the last few years. New sections have been incorporated on many subjects, including, among others, the effect of hypertonic saline on intracranial pressure, the relation of the hypothalamus to the pituitary, the dietetic treatment of pernicious anæmia, Sabin's work on the development of the blood, the coronary circulation in health and disease, and Lewis's recent work on the "H" substance. Several sections have been rewritten; among which are those dealing with fat metabolism and the output of the heart, and many new illustrations have been added, thus greatly enhancing the value of the text. For those unacquainted with the "old terminology" the B.N.A. equivalents are placed in the text in brackets and a glossary of equivalents has been added. The book has been well produced. It should prove popular, not only with students preparing for examinations, but also with clinicians; for, after all, the only firm basis for a good understanding of pathological processes is a sound knowledge of the normal functions of the body. This the book provides, and also points out the clinical application of the ascertained facts to the elucidation of diseased states.

**Outlines of Dental Science. XII.: Orthodontics.** By A. G. WILSON, L.D.S. Pp. xii., 168. Illustrated. Edinburgh: E. & S. Livingstone. 1928. Price 8s. 6d.—This little book is one of a very interesting series published recently. Each volume is complete in itself, dealing as it does with one particular subject in the dentist's art. The whole series is necessary for a library. The subject-matter of this volume deals with the complicated and fascinating subject of irregular jaws and teeth in children. The author has stated the case plainly, and in a manner very concise and easily understood. The American view is clearly expounded in a way that should do much to help in clearing away many of the points that the average student in an English dental school finds so difficult.

**Erythema Nodosum.** By J. ODERY SYMES M.D. Bristol: John Wright & Sons Ltd. 1928. Pp. 72. Price 5s.—Within a small space Dr. Symes has compressed the fruit of years of observation. The account that is offered here is fuller in many details than the Long Fox Memorial Lecture published in this issue, and in fact furnishes a complete description of the disease. Both in manner and in matter it is a production of which the author and the publishers may well be proud. Dr. Symes presents a study that, for thoroughness, lucidity and balance, is a model of what a monograph should be.

**Insomnia and Drug Addiction.** By P. C. C. FENWICK. L.M.S.S.A. Pp. xii., 56. London: H. K. Lewis & Co. Ltd. 1928. Price 2s.—This little monograph deals very clearly and succinctly with the subject of drug-habit, giving a list of these substances for which habit is acquired and setting out the causes, symptoms and effects of drug addiction. The subject is one very inadequately dealt with in the ordinary text-book, and to the general practitioner, especially if he be a beginner, the chapter on treatment will be invaluable, for it details the hyoscine treatment of morphinism and the gradual reduction cure as applied to cases of addiction to morphia, cocaine and alcohol. The chapter on after-treatment is full of practical common-sense hints.

**Gastro-intestinal Diseases.** Edited by D. WATERSTON, M.D., F.R.C.S. Pp. viii., 278. Illustrated. London: Oxford University Press. 1928. Price 10s. 6d.—This volume is composed of lectures delivered at the Mackenzie Institute. The views expressed by the various writers are lucid and up to date, and the book should be a very valuable addition to the library of the clinician. As regards the ocular manifestations which may occur in gastro-intestinal disorders, a very diverse series is presented. There is an interesting reference to the causation of hunger and appetite, and an illuminating discussion on the movements of the stomach and their relationship to peristalsis in other parts of the alimentary tract; the converse, abnormalities in colon movement and the effect of this upon gastric function, is well remarked upon, as well as the influence of psychic states. The clinician will find much help from the lecture on hyperæsthesia and referred pain; the investigators have located well-defined areas of hyperalgesia and hyperæsthesia in affections of the gall-bladder and appendix, but not, apparently, in cases of diseases of the stomach and duodenum. Dealing with the dyspepsias, recognition is made of hypersthenic and hyposthenic gastric states; the influence of psychic conditions on gastric disorders is elaborated at some length: "the function of digestion must be regarded as . . . a sensitive point in a complex psychic and nervous organisation." The diagnosis of gastric and duodenal ulcer is handled on common-sense grounds: a good clinical history still holds sway; X-rays are not by any means always to be relied upon. Hæmatemesis and gastrostaxis are discussed at some length. A hint is thrown out as to the selective affinity of organisms of streptococcal type for the gall-bladder, and the origin of duodenal ulcer by infection from this site. There is a good series of photographs of X-ray findings in carcinoma of the stomach; much stress is laid on peristaltic interference as seen on barium investigation, and it is claimed that it probably preceeds any clinical symptom. This is a praiseworthy collection of the views of different writers, fully maintaining the high standard of clinical work set by the founder of the Institute.

**The Robert Jones Birthday Volume: A Collection of Surgical Essays.** By Various Authors. Pp. xii., 434. 355 illustrations. London: Oxford University Press. 1928. Price 42s.—This volume represents a really great tribute to a really great man. It is certainly not within our recollection that any British surgeon has ever had such a tribute paid to him, although the practice of presenting a "festschrift" to a great teacher is a well-known practice on the Continent. Opening with a most eloquent preface, which is as affectionate as it is sincere, by Moynihan, it is continued by twenty-four essays on subjects relating to bone and joint surgery by men in all parts of the world. Many of these have been both pupils and assistants of Sir Robert Jones; others are leaders in orthopædic surgery in America, Canada, France, Italy and Holland. It is quite impossible in the limits of an ordinary review to do anything like justice to the various essays, but at the risk of invidious distinctions one may point to some of the outstanding papers. Putti gives a most interesting account of two cases cf tumour of the femur and the ingenious method by which he made good the bone after the removal of the growths. Murk Jansen of Leyden gives a characteristically learned discussion on the dissociation of bone growth. Elmslie adds to his own reputation and to our knowledge of fibro-cystic diseases of bone by a paper which is an amplification and a continuation of a classical one he wrote in 1914. Holland, from his past experience, gives an account of the accessory bones of the foot in a paper which will be of great value for the purposes of reference. Platt deals with the nerve complications of injuries round the elbow-joint, and gives such a large number of cases that it is possible to draw valuable conclusions as to prognosis and treatment. Bristow has added to our knowledge of the cysts of the semi-lunar knee cartilages by a description of eleven new cases, nine of which affected the external meniscus. He regards them as degenerative in origin and comparable to the ganglia of tendon sheaths. Naughton Dunn summarises his experience of the stabilising operations for flail foot introduced by himself some years ago. We can say without any fear of contradiction that nearly all the papers contained in this memorial volume represent real additions to our knowledge of orthopædic surgery. The appearance of the volume is an eloquent testimony to the great influence that Sir Robert Jones still exercises over thought and progress.

**Requisites and Methods in Surgery.** By C. W. CATHCART, C.B.E., F.R.C.S., and J. N. J. HARTLEY, O.B.E., F.R.C.S. Pp. viii., 476. 246 illustrations. Edinburgh: Oliver & Boyd. 1928. Price 12s. 6d.—This volume is written on much the same lines as other works on minor surgery for the student and house surgeon. It cannot be regarded as a possible addition to the student's library, but rather as another book in the vast library from which he has to choose. It has, however, several features which might commend it, and which might justifiably turn the balance in favour of its special selection. The chapter on case taking is very comprehensive, and includes a description of the methods of examining the urine for possible abnormal constituents. A short account of cystoscopy and catheterisation of the ureters is given, and a few hints on the use of barium and X-rays as an additional method of aiding diagnosis. The chapter on anæsthetics is very helpful and that on massage is a welcome inclusion. The question of treatment is brought well to the fore, and even the failure of any given method is anticipated by a description of post-mortem examination and the medico-legal aspects of death. Hospital economy is another somewhat unusual feature of the book, and a chapter on trusses and artificial limbs, an appendix including a description of the clockwork control of Carrel-Dakin and hints on the index of cases under diseases concludes the volume. The above does not, by any means, include the total sphere of usefulness of the work; a vast amount of information is included between its covers which might more than repay the owner his twelve shillings and sixpence.

**Extra Pharmacopœia, Vol. I.** By MARTINDALE and WESTCOTT. Nineteenth Edition. Revised by W. H. MARTINDALE, Ph.D. Pp. xxxvi., 1,207. London: H. K. Lewis & Co. Ltd. 1928. Price 27s. 6d.—During the forty-five years which have elapsed since the publication of this work it has been a constant friend to vast numbers of medical practitioners and pharmacists. The increasing number of medicinal preparations which become available as time goes on has rendered it necessary to enlarge this work nearly three times. We have constantly referred to it for over thirty years, and have never been disappointed. It is impossible to mention all the good points of this work, but an outstanding feature is the inclusion of a great number of references to researches by various workers in the different branches of materia medica. The authors have always succeeded in keeping the work up to date, which entails an extraordinary amount of care and reading. We need only say that it should be included in the library of every medical practitioner and pharmacist.

**A Handbook for Nurses.** By J. K. WATSON, M.D. Eighth Edition. Pp. xii., 898. Illustrated. London: Faber & Gwyer Ltd. 1928. Price 8s. 6d.—We are always pleased to welcome a new edition of this valuable *Handbook for Nurses*, and we are grateful to the author for giving us yet another. The additional 100 pages contain a great deal that is useful, and we especially like the chapter on mental diseases. The increased number of illustrations is a great asset, especially those of the skin diseases. The book is a complete summary of the most important things a nurse needs to know; it will guide her throughout her training and final State Examination, and provide her with information of and treatment for practically any emergency which may occur in hospital or private work.

**Dental Medicine.** By F. W. BRODERICK, M.R.C.S., L.R.C.P., L.D.S. London: William Heinemann Ltd. 1928. Pp. xviii., 364. Price 21s.—The title that Mr. Broderick has selected for his book suggests that there is more in the speciality of dentistry than the repair and replacement of teeth, that, in addition to this being a specialised branch of surgery, the science and art of medicine should be utilised in practice. The writer shows how, in the attempt to compensate a change in the acid base equilibrium of the blood, certain ill effects are brought about in other tissues that will, as the result of natural bio-chemical laws, bring about changes in the teeth which will result finally in those conditions that we call dental caries and pyorrhœa. Stated briefly, his theory as to the origin of the former condition is that, as the result of an attempt to compensate an acidosis, there arises an alteration in the reaction of the saliva towards the acid side of the normal, that when this becomes more acid than the iso-electric point of the proteins which that saliva contains, there takes place a passage of calcium ions from the teeth to the saliva, which in time will lead to the disintegration of the dental enamel and finally the formation of a cavity. Pyorrhœa alveolaris, on the other hand, according to his view is brought about by an opposite state of things. Here a threatened alkalosis in compensation results in the laying down of subgingival tartar, which, through the irritation which it brings about in the subgingival trough, is the direct cause of the clinical signs, the pocket and the alveolar destruction, which are the main features of that condition. As a secondary effect of the attempt to compensate the alkalosis, conditions as to salivary reaction will result which will be opposite to those which he blames for the production of caries, and the teeth will become hypercalcified and of extreme hardness and incidentally immune from caries. By a careful classification of various forms of gingivitis Mr. Broderick separates those which are the precursors of pyorrhœa from those which are not, and in so doing removes the difficulties which have been present in the minds of many men in accepting his dictum that dental caries and pyorrhœa are in reality opposite and antagonistic conditions. Chapters are included on such matters as the reaction of the blood and the manner in which this is kept stable ; the result of changes or tendencies to change in this reaction, and the clinical conditions known as acidosis and alkalosis ; endocrinology ; the metabolism of lime salts ; and the physiology of the saliva and the circumstances which tend to alter the reaction of that secretion. As a result of the visualisation of the conditions on which the ætiology of dental caries and pyorrhœa primarily rests, which the author here presents, he formulates a preventive treatment which becomes scientific and rational, by which the causes of the conditions may be removed or, at any rate, prevented from compensating in such a manner as to be a danger to the teeth and their supporting tissues. Altogether this is a most fascinating and stimulating book, which, going as it does to the very origin of diseased conditions, should be read by all students of disease. Practitioners of every branch of medicine will find in it much of interest.

